# Employee Political Skill, Supervisor-Subordinate Guanxi, and Work-Family Conflict: The Cross-Level Moderating Role of Family-Friendly Practices

**DOI:** 10.3390/ijerph17145185

**Published:** 2020-07-17

**Authors:** Le Tang, Rentao Miao, Lai Jiang

**Affiliations:** 1 School of Labor Economics, Capital University of Economics and Business, Beijing 100070, China; mrtmiao@hotmail.com; 2 Periodical Head Office, Capital University of Economics and Business, Beijing 100026, China; jianglai@cueb.edu.cn

**Keywords:** political skill, supervisor-subordinate Guanxi, work-family conflict, family-friendly practices, moderated mediation model

## Abstract

Many studies have examined the negative effects of work-family conflict on society, organizations, and individuals. Nonetheless, alleviating employee work-family conflict is a topic worthy of further investigation. Few studies examine the antecedent variables of work-family conflict from personal skill and Guanxi perspectives. Studies that test the moderating role of family-friendly practices at the organization level are also rare. Accordingly, we collected data from 404 employees of 51 organizations. The research data are time-lagged and multileveled. The results of hierarchical linear model (HLM) show: (1) Employee political skill negatively affects employee perceived work-family conflict; (2) Employee political skill positively affects supervisor-subordinate Guanxi; (3) Supervisor-subordinate Guanxi partially mediates the relationship between employee political skill and employee work-family conflict (that is, employees can use their political skill to build high-quality supervisor-subordinate Guanxi, further reducing their perceived work-family conflict); (4) Organization family-friendly practices negatively moderate the relationship between supervisor-subordinate Guanxi and work-family conflict (that is, in organizations with high level family-friendly practices, the negative relationship between supervisor-subordinate Guanxi and work-family conflict becomes weak); Furthermore, by coding with Mplus software (Muthen & Muthen, Los Angeles, CA, USA), we also find: (5) Organization family-friendly practices moderate the indirect effect of employee political skill on employee work-family conflict. The results have both theoretical and empirical implications. Further research directions are addressed at the end.

## 1. Introduction

Work and family are the two very important aspects of individuals’ social life. However, employees often harm the interests of their families when striving for career development, causing conflict between work and family. Although that conflict is bidirectional (i.e., Work-to-Family Conflict and Family-to-Work Conflict), previous research shows that individuals’ perceived Work-to-Family Conflict is three times greater than Family-to-Work Conflict [1]. As a result, scholars pay more attention to Work-Family Conflict (WFC). Many studies have examined the negative effects of WFC on society, organizations, and individuals. For instance, WFC significantly reduces women’s fertility intentions [2], has a significant negative impact on employees’ job performance [3], causes workers’ job burnout [4], increases employees’ turnover intentions [5], and negatively impacts firm performance [6]. Moreover, WFC is associated with lower levels of general health [7] and can reduce individuals’ life satisfaction [8] and subjective wellbeing [9]. Integrating and balancing work and family demands represent the most severe challenges for organizations, families, and individuals [10]. Therefore, further studies on how to effectively reduce employees’ WFC will be a meaningful topic.

Many antecedent variables alleviate individuals’ WFC. Lapierre et al. summarized the antecedent variables affecting WFC through meta-analysis, including two main categories: work factors and individual factors [11]. From the perspective of individual factors, the most studied demographic variable is gender [12]. However, gender caused WFC is not obvious [13,14]. According to role theory, individuals need different skills when they play different roles. Conflicts between roles may arise due to the lack of skills. Jin et al. showed that individuals’ coping skills, such as rational action and positive thinking, could alleviate their perceived WFC [15]. But, unfortunately, there are few studies on alleviating WFC from the perspective of individuals’ skills. As an effective social skill, political skill (PS; the term political skill in this study is a kind of social skill and has nothing to do with national, party or personal politics) has proved able to relieve workplace stress [16] and reduce role conflict [17]. Whether it can also alleviate WFC is worth discussing.

Furthermore, it is rough to simply link PS and WFC. Based on work/family border theory, the relationship between supervisor and subordinate may influence subordinate’s WFC [18]. In fact, employees are not passive when they interact with their supervisors. They can build a good relationship with supervisors through their skills. However, supervisor-subordinate Guanxi (SSGX; Guanxi is a special type of relationship generated from Chinese contexts) in Chinese organizations differs from Leader-Member Exchange (LMX) generated in Western contexts. SSGX in China covers mainly non-work exchange [19] and is the extension of family relations [20]. Tang et al. find that employees could use their PS to build good SSGX and further increase their promotion probability [21]. Meanwhile, previous studies show that family-supportive supervisor behaviors have a significant negative effect on employee WFC [22]. Therefore, SSGX may become the bridge between employee PS and WFC in Chinese organizations. Thus, exploring the relationship between employee PS and WFC from Guanxi perspective has great significance, both in a theoretical and practical sense.

Although good SSGX can alleviate employee WFC, finding the boundary conditions have more practical significance. The essence of the work-family relationship is the relationship among individuals, families, and organizations. Work-family balance can benefit not only employees but also organizations [23]. Therefore, the role of the organization is indispensable in alleviating employee WFC. In fact, scholars began to research human resource practices that can promote work-family balance as early as the 1970s, when facing the reduction in the labor force due to fertility decline [24]. Organizations provide such family-friendly human resource practices to help employees integrate work and family roles [25]. So, will Family-Friendly Practices (FFPs) become the boundary condition of the mechanism PS—>SSGX—>WFC as an organizational factor? To be specific: In organizations with high level FFPs, is the negative effect of SSGX on WFC as strong as in organizations with low level FFPs? This paper answers that question.

In summary, from the perspective of skill and Guanxi, this study first tests the negative effect of employee PS on WFC; second, it tests whether employees can build good SSGX with their supervisors through their PS; third, it further explores the mediating role of SSGX between employee PS and WFC; fourth, it introduces FFPs into the model as an organizational variable and explores the cross-level moderating effect of FFPs between SSGX and WFC. Finally, the paper examines the moderated mediating effect of FFPs in the PS—>SSGX—>WFC mechanism.

## 2. Theoretical Background and Hypotheses

### 2.1. Employee Political Skill and Work-Family Conflict

Organizations are political arenas. Political behaviors exist because they may have the power to influence others’ attitudes and behaviors to protect one’s own interests [26]. Moreover, to play a role in this competitive environment, organization members need not only the political will to engage in political behavior but also PS to develop effective political behavior. However, invalid and untimely political behaviors often lead to bad, even malignant consequences [27]. Therefore, people tend to associate “politics” with negative impressions, such as behind-the-scenes manipulation, self-interested behaviors, favoritism, and irregularities. Why do individuals’ political behaviors fail in the organization? If an individual wants to succeed in a competitive and changeable organization environment, he/she must have the corresponding PS [28]. PS is a kind of social skill that can help individuals better judge situations and perform appropriate political behaviors to achieve individual or organizational goals [29].

Ferris et al. define PS as “the ability to effectively understand others at work, and to use such knowledge to influence others to act in ways that enhance one’s personal and/or organizational objectives,” described in four dimensions [30]. First is social astuteness; individuals processing social astuteness can accurately interpret others’ behaviors and understand the organization’s climate, which helps them keenly adapt to diverse social situations. The second dimension is interpersonal influence; individuals skilled in interpersonal influence can exert a powerful influence on others in a subtle way in different contextual conditions, to achieve their goals. The third dimension is networking ability, which refers to being adept at building, developing, and using diverse social networks and benefiting from them. The fourth dimension is apparent sincerity, which refers to high levels of sincerity, authenticity, integrity, and genuineness that individuals display to others. PS seems to be similar to other social effectiveness constructs, such as self-monitoring [31] and emotional intelligence [32]. However, PS specifically refers to social interaction skills at work which make it conceptually different from others. Also, previous empirical studies have proved that PS is different from other constructs [30,33,34].

According to role theory, people play two different roles at work and at home. Lacking skills may lead to conflicts between those roles [35]. Using meta-analysis, Lapierre et al. point out too much job stress or lack of work support are important causes of WFC [11]. However, employees’ coping skills can help them to reduce WFC by making plans, setting priorities, and seeking help [36]. Moreover, individuals’ coping strategies, such as rational action and positive thinking, can alleviate individuals’ WFC [15]. As an effective social skill, PS can also help employees relieve work pressure, obtain work support, and reduce their perceived WFC. Employees possessing PS can use their excellent interpersonal influence and extensive social network to get more support and resources to reduce their perceived work pressure [16]. As a personal resource, PS can also reduce job tension [37]. Employees with high-level PS have strong powers of discernment and self-awareness. They know how to use the information they get and their interpersonal skills to influence others’ behaviors and decisions, which can help them reduce role pressure caused by uncertainty when they interact with others [38]. Facing workplace pressure, they are more confident and calmer than employees with low-level PS [39]. PS can also reduce the negative spillover of work stress, further reducing employee perceived role conflict [40]. Employees with negative emotions report higher levels of WFC [41]. However, PS can effectively reduce employees’ negative attitudes and behaviors, helping them to acquire more positive feelings [42]. Finally, many studies show that supervisors’ family support can reduce employee WFC [43], and highly politically skilled employees are good at getting support from others. Thus, we hypothesize the following:

**Hypothesis** **1:** *Employee political skill has a significant negative effect on employee work-family conflict.*

### 2.2. Employee Political Skill and Supervisor-Subordinate Guanxi

Employees are not passive when interacting with their leaders. They can show active and positive attitudes and behaviors to change leaders’ cognition, promote exchange between the two, and build a good relationship [44]. However, cultural settings influence and restrict relationships or Guanxi (a special type of relationship generated in the Chinese context). Different from LMX that originated in Western culture, Guanxi between the supervisor and the subordinate in an Eastern context stresses sentiment (Qing, human feeling) and unconditional loyalty (or obligations), which are the core elements of family relations [45]. Therefore, LMX, which is limited to the scope of work, cannot reflect the unique meaning of Guanxi between leaders and followers in Chinese organizations. Law et al. (2000) point out, “SSGX in China covers mainly non-work exchange within the vertical dyad and the benefits being exchanged can be social and economic in nature,” making SSGX significantly different from LMX [19]. Based on family collectivism, Chen et al. (2009) state that Guanxi between the supervisor and the subordinate in Chinese culture is a kind of integration and extension of the family relationship, namely, a “family-oriented working relationship.” SSGX is an emotional and personal connection between supervisors and subordinates [20].

Although LMX and SSGX both originate from social exchange theory, they are different in exchange scope, exchange content, and status of supervisor and subordinate, which causes them to adopt different norms of reciprocity [34]. Since understanding the theoretical characteristics of SSGX is the key to explaining its formation, we summarize the differences between LMX and SSGX in Table 1.

Based on these three characteristics, we believe that PS possessed by employees can effectively help them build good SSGX. First, SSGX forms mainly after work. Chen et al. (2009) point out that such interactions as “visit each other during holidays” or “help supervisor deal with some family errands” can help employees build and maintain good SSGX [20]. Therefore, employees must spend extra time and effort to build and maintain good Guanxi with their supervisors [19], exactly what characterizes employees with strong networking ability. Individuals possessing networking ability attach great importance to network resources, and they also excel at building good personal relationships with others and keeping in touch with important people [46]. They spend time and effort on interpersonal communication, including with the supervisors who closely relate to their work and life [47].

Second, the basis for establishing SSGX is emotional factors. Leaders have limited time and energy, so not every subordinate has the opportunity for in-depth interaction with leaders [48], especially emotional communication. Employees must understand the demands of leaders and know their preferences in order to gain their trust and become their leaders’ “in-group” members [49]. This characterizes socially astute employees. In supervisor-subordinate interaction, the leader’s needs are often not publicized. Employees high on social astuteness can quickly and accurately understand the leader’s intentions through keen observation [50]. Moreover, employees with strong abilities in interpersonal influence have good communication skills [51]. As Chen et al. (2009) point out, they can build high-quality SSGX through emotional resonance with leaders by “sharing thoughts, opinions, and feelings toward work and life” [20].

Third, subordinate loyalty is the key to building and maintaining close SSGX. Chinese leaders attach great importance to the “loyalty” of their subordinates. Zheng (1995) argues that Chinese leaders will judge Guanxi with their subordinates according to three aspects: “loyalty”, “affinity,” and “talent.” If subordinates are not loyal enough, the leaders will regard the "talented" ones as an object of precaution and classify the “untalented” ones as marginal individuals, and they are both become the leader’s "out-group" members [52]. However, employees possessing interpersonal influence can establish an obedient and loyal image with their leaders [53]. Cheng and Wang (2015) point out that “in Chinese organizations, leaders often play the role of authoritative ‘father’, and employees with high interpersonal influence can show a highly obedient ‘child’ image when interacting with their supervisors through flattery and other subtle ways” [54]. The matching roles of “father” and “child” will stimulate high-quality Guanxi between supervisor and subordinate [20]. In addition, employees with apparent sincerity are more likely to gain the trust of their leaders [55]. Employees hide their self-interest motivation by showing sincerity, so their supervisors have a benign expectation of their future behavior, which improves supervisors’ perception of subordinates’ loyalty [56], thus building intimate Guanxi between them. Therefore, employees are not passive in the process of building Guanxi with their supervisors. Politically skilled employees can adopt active and positive ways of changing a leader’s attitudes and behaviors and building good SSGX [57].

**Hypothesis** **2:** *Employee political skill has a significant positive effect on supervisor-subordinate Guanxi.*

### 2.3. The Mediating Role of Supervisor-Subordinate Guanxi

Work-family conflict was first called work-family interference which was proposed by Kahn et al. (1964) based on role theory [58]. They note that work-family interference is a type of role conflict. According to role theory, people play two different roles at work and in a family. Lack of experience or conflict of responsibilities causes role conflict, which makes individual difficult to succeed in all roles [59]. According to “family-like” theory, Guanxi between supervisors and subordinates in the Chinese context is the extension of a family relationship with the characteristic of obligation [20]. In Chinese organizations, paternalistic leadership is the dominant leadership style. Guanxi between supervisors and subordinates outside of work is similar to that between “father” and “child” in the family [60]. In close SSGX, the consistency of identity cognition of “good father” and “good child” can help employees transfer roles smoothly, helping them to reduce role conflict between work and family [61].

Second, according to work-family spillover theory, individuals bring their attitudes, emotions, and behaviors generated at work into their family field. These attitudes, emotions, and behaviors can be positive or negative, and WFC is the product of negative spillover [62]. Lacking work support, experiencing excessive work pressure and negative emotions all lead to negative spillover causing WFC [63]. Previous studies show that family supportive supervisor behaviors in daily management can effectively help employees alleviate their WFC [64]. These behaviors not only help employees in their work field but also provide support for fulfilling their family responsibilities, which are the key factors in helping employees balance work and family [65]. However, supervisors’ time and resources are limited, so they cannot provide all their subordinates with the same level of help and support [66]. Especially in Chinese organizations with differential Guanxi patterns, supervisors tend to build different levels of Guanxi with different subordinates. Then, supervisors provide help and support to subordinates according to the intimacy of SSGX [67]. Employees who have built good SSGX will receive extra care, help, and resources beyond the principle of fairness, not limited to work and expanding outside of the workplace [34]. The important identity symbol of “insiders” plays a key role in Chinese society, which attaches great importance to human feelings and relations. Good SSGX can help individuals get more formal and informal support [19]. Leaders will not only provide more resources to close subordinates, helping them reduce their professional pressure, but will also be more tolerant of them, reducing their negative emotions [68] thus helping employees alleviate WFC.

Third, Clark (2000) proposes work/family border theory, showing that individuals live in two important domains, work and family, and respectively connect with different domain members and rules. They are daily border-crossers between the two domains [18]. The main reason to keep work-family balance is the relationship between border-crossers (individuals who frequently transit between work and family domains) and border-keepers (domain members who have special influence in defining the domain and its border). Clark’s theory includes two important propositions. First, “border-crossers whose domain members have high other-domain awareness will have higher work-family balance than border-crossers whose domain members have low other-domain awareness.” Second, “border-crossers whose domain members show high commitment to them will have higher work-family balance than border-crossers whose domain members have shown low commitment to them.” At work, employees’ most influential border-keepers are their direct supervisors. Based on the propositions above, good SSGX will help employees reduce work-family conflict. First, supervisors and subordinates in good Guanxi will have more interactions and communications, like having dinner together or visiting each other on holidays. So, they have more emotional communication and become familiar with each other’s family members and duties. It can help supervisors better understand employees’ family domain and increase the consistency of the definition of the work-family border [69]. Since the main reason for WFC is the inconsistency of work-family border [70], good SSGX will certainly help employees reduce their WFC. Second, mentioned above, paternalistic leadership is the dominant leadership style in China [60]. In close SSGX, leaders play the role of “good father,” which makes them feel obligated to their subordinates. They will provide conveniences for their subordinates in both work and life. They understand the responsibility of their subordinates’ family and regard the tolerance and support for their subordinates as a kind of commitment. For example, when employees ask for leave for personal affairs, supervisors can understand and provide corresponding help, which can alleviate employees’ WFC.

In summary, as the agent of the organization, leaders interact with employees in daily work, formally and informally performing management functions on behalf of the organization. They have the power to allocate tasks, evaluate performance, and provide resources to their subordinates, which gives them significant influence over their subordinates’ development in the organization. Therefore, leaders play an indispensable and important role in the process of reducing employee WFC. Employee PS must influence WFC through SSGX:

**Hypothesis** **H3:** *Supervisor-Subordinate Guanxi has a significant negative effect on employee work-family conflict.*

**Hypothesis** **H4:** *SSGX mediates the relationship between employee political skill and work-family conflict.*

### 2.4. The Cross-Level Moderation Role of Family-Friendly Practices

Although we explored the negative effect of SSGX on WFC in H3, it is unclear under what kind of circumstances its effects may be more or less pronounced. Therefore, in this study, we propose that one such circumstance is the level of FFPs offered by organizations. The essence of work-family relationship is the relationship among individuals, families, and organizations [71]. Work-family balance can benefit not only employees but also organizations [23]. Therefore, the role of the organization is indispensable in alleviating employee WFC. FFPs are a set of formal programs developed by organizations, aiming to provide support to employees in order to balance their family and work demands [72]. Previous studies show that employee perceived FFPs can directly or indirectly reduce WFC [73]. However, as a type of human resource practice at the organizational level, FFPs have a certain stability and unity [74]. Employee perceived FFPs may be subjective since many factors can influence them, such as employee demographic characteristics [75]. Therefore, exploring the influencing mechanism of FFPs from the organization level is more objective and practical.

Supervisors have certain discretion over the type and level of family support that their subordinates receive, regardless of whether the organization provides FFPs [76]. When organizations provide little (or no) FFPs, supervisors become the only source for employees to reduce WFC. However, employee perceived WFC will not disappear innocently. The lack of official FFPs makes supervisors’ family supportive behaviors more important [64]. SSGX in China implies “differential pattern” and “authority orientation” [77]. How supervisors allocate resources and make decisions depends more on the intimacy of the relationship. Especially when the formal system of an organization is invalid or imperfect, SSGX will become the substituted mechanism of the formal system of an organization and dominate employee career development [78]. Therefore, in the absence of formal FFPs, SSGX plays a more important role in reducing employee WFC. In Chinese organizations, “rule by man” and “rule by law” play alternate roles in various fields. The lack of formal organizational procedures makes relationships more important [79]. That is, when organizations provide few FFPs, the negative effect of SSGX on employee WFC is stronger. Furthermore, regarding H4, employees use their PS to build high-quality SSGX and reduce their WFC. When family supports from supervisors sharply contrast with the lack of FFPs in the organization, supports from the supervisors for subordinates’ family demands become more prominent [80]. Moreover, since supervisors’ family support behaviors are not officially approved, employees may have a stronger positive perception of their supervisors’ family-tolerant attitude [81]. In this situation, employees are more likely to use their PS to build and maintain good SSGX, and the indirect negative effect of SSGX on WFC becomes stronger.

In contrast, when the level of FFPs is high, employees can get family supports not only from their supervisors but also through the organization’s official channels [80]. That is to say, the substitution of FFPs weakens the effect of SSGX on WFC. In other words, when organizations provide enough supports to meet the needs of employee work-family balance, employees will preferentially choose to use formal FFPs to reduce their WFC, without relying on their supervisors. Supervisors’ family support may have less impact on WFC because it is less salient when support also comes from organizations, weakening the negative effect of SSGX on employee WFC. Moreover, when the level of FFPs is high, employees may attribute support from their supervisors to the broader effort of the organization [82]. This is consistent with the concept of leadership substitution, which refers to factors that reduce the impact of leaders’ behavior [83]. The benefits that employees get from the organization can neutralize (moderate) the effects of leader behavior. Specifically, in the leadership substitution literature, organizational reward is an important moderator between leadership behavior and outcome variables [84]. Therefore, we propose that when organizations provide high levels of FFPs, the negative effect of SSGX on employee WFC becomes weaker. Furthermore, we combine the mediation effect and moderation effect, testing the moderated mediation model of this study. When organizations’ FFPs are high, the negative relationship between SSGX and WFC is weaker, thus, the effect that employee PS can have on WFC will be less transmitted through SSGX. Based on this discussion, we hypothesize the following:

**Hypothesis** **5:** *The organization level FFPs moderate the negative relationship between SSGX and employee WFC in such a way that the relationship is stronger when FFPs are low, than when they are high;*

**Hypothesis** **6:** *The organization level FFPs moderate the mediation effect of SSGX between employee political skill and WFC (Hypothesis 4) in such a way that the mediation effect is stronger when FFPs are low, than when they are high.*

**Figure 1:**
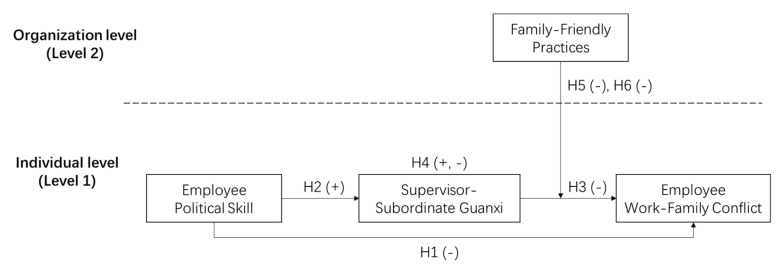
Research model.

Based on these discussions, Figure 1 shows the research model of this study.

## 3. Method

### 3.1. Participants and Procedure

To verify the theoretical hypotheses above, we collected data from 51 organizations in China, including public institutions, state-owned companies, foreign companies, and private companies, covering industries like education, energy, communications, and consulting. To ensure the diversity and randomness of the sample, the researchers attempted to reach as many organizations as possible. The questionnaires were distributed and recovered through each organization’s contact person in the human resources department. To avoid common method biases, the data collection occurred in two rounds with an interval between them of one month. Specifically, the first round of data collection occurred in mid-May 2019. After communicating with the contacts, we estimated the sample size of each organization in advance and mailed the questionnaires to the contacts, with 10 or 15 employee questionnaires for each organization. A total of 565 employee questionnaires were sent. In the first round of data collection, employees were asked to evaluate their own PS and Guanxi. Meanwhile, to collect data on the organization level FFPs, we also issued an FFPs questionnaire for each organization, which was completed by the HR department’s time or benefits management specialist, at the invitation of the contact person. The first round of data collection lasted for seven days. A total of 451 employee questionnaires were collected with a response rate of 79.82%, while 51 organization questionnaires were collected with a response rate of 100%. The second round of data collection occurred at the end of June and lasted for 10 days. Data were collected anonymously, but to match the two rounds of data, the serial numbers were printed on the bottom of each questionnaire. The contacts recorded the name of each employee corresponding to each serial number. Therefore, the contacts carried out the second round of questionnaire distribution based on the list of participants in the first round distribution. During the second round survey, employees were asked to rate their own perceived WFC. A total of 451 employee questionnaires were issued and 418 questionnaires were recovered with a response rate of 92.68%. Additionally, we asked each organization’s contact person whether there was any change in FFPs during this survey interval, to judge the necessity of re-collecting FFPs data. Fortunately, no organization changed its FFPs during this period.

In summary, 565 employee questionnaires and 51 organization questionnaires were distributed in this survey. A total of 418 employee questionnaires were recovered. Relationship building takes time even for employees with high PS. Hence, we removed the data of which cooperation periods between supervisor and subordinate are less than 12 months in the final sample to represent stable SSGX. The final number of valid employee questionnaires was 404 with a response rate of 71.50%. The characteristics of the valid employee sample (*N* = 404) appear in Table 2. Also, we recovered 51 organization questionnaires with a response rate of 100%. To avoid excessive costs of questionnaire distribution, we communicated with each organization’s contact person in advance to ensure the effective acquisition of organization level data. Benefiting from our professional background – human resource management, the contacts in each organization are either our previous classmates, colleagues or recommended by them. The close ties resulted in a high response rate. In the valid organization sample (*N* = 51), 15.7% were public institutions, 45.1% were state-owned companies, 19.6% were foreign companies, and the remaining 19.6% were private companies. Large-scale organizations with more than 1000 full-time employees represented 56.9%, 35.3% were organizations with 300–1000 employees, and 7.8% were organizations with less than 300 employees. Among the 51 organizations, the sample size of employees in each organization is 6 to 14. The proportion of organizations with 6 to 9 employees reached 82.4%, and the average employee sample size of all organizations was 7.92 (SD = 2.077). 

### 3.2. Measure

All responses were collected using a 5-point Likert scale from “1” strongly disagree to “5” strongly agree. Since the target samples were Chinese, the scales used to measure these variables were either developed in Chinese or showed excellent psychometric properties in the Chinese context. The original scales of Political Skill, Supervisor-Subordinate Guanxi, and Work-Family Conflict were developed in English, so we implemented a parallel, double-blind "translation-back translation" procedure to ensure conceptual consistency. We also communicated with HR specialists of several sample organizations to make sure that the questionnaire was easy for the staff to understand.

**Political skill.** Employees rated their own PS by using Ferris et al.’s (2005) 18-item scale [30]. The scale includes four dimensions: networking ability (with a sample item, “I spend a lot of time and effort at work networking with others”), interpersonal influence (with a sample item, “I am able to make most people feel comfortable and at ease around me”), social astuteness (with a sample item, “I am particularly good at sensing the motivations and hidden agendas of others”), and apparent sincerity (with a sample item, “When communicating with others, I try to be genuine in what I say and do”). The PS scale showed a high level of alpha reliability (α = 0.882).

**Supervisor-Subordinate Guanxi.** This variable was measured by the six-item scale developed by Law et al. (2000) [19]. No repetition appears in the definition compared to LMX, since SSGX measures the relationship built outside the workplace. Employees were asked to report their perceived Guanxi quality with their supervisors. Sample items are “During holidays or after office hours, I would call my supervisor or visit him/her” and “I always actively share with my supervisor about my thoughts, problems, needs and feelings”. The SSGX scale showed fair reliability (α = 0.755).

**Work-family conflict.** Employees rated their perceived WFC level using Frone and Yardley’s (1996) six-item scale [85]. Sample items are “After work, I come home too tired to do some of the things I’d like to do” and “My work takes up time that I’d like to spend with family/friends”. The alpha reliability of this scale is 0.705, which is acceptable. 

**Family-friendly practices.** This variable was measured by Zhao et al.’s (2019) 18-item Work-Family-Balance Human Resource Practice scale, which was developed in the Chinese context [86]. One HR professional from each organization rated his or her organization’s FFPs from the organization level. The scale includes four dimensions: flexible working schedule (with a sample item, “Employees in the organization can work from home or work remotely”), family care (with a sample item, “Our organization provides family trip or organize family actives for employees”), support to employee (with a sample item, “Our organization often provides conflict handling related training for employees”), and vacation provided (with a sample item, “Our organization provides unpaid leave for employees”). Prior studies with Chinese samples have shown good reliability of this scale. Alpha reliability of the scale in this study is 0.957.

**Control variables.** In this study, we controlled both individual and organizational characteristics that affect the research model. Employee’s education level may have had a positive effect on PS [33], while employee gender, age, marriage status, and having children under 18 may influence WFC [59]. Furthermore, relationship building takes time. Cooperation period between supervisor and subordinate may affect the quality of SSGX. A subordinate and his/her supervisor need time to interact to understand the other’s needs. SSGX between the two becomes stable with the increase of cooperation and interaction over time [19]. Furthermore, WFC is likely to be influenced by the characteristics of the organization such as size and property [87]. Large organizations with a strong human resource management track record were more inclined to offer a wider variety of FFPs [88]. Public sector organizations were more predisposed to provide FFPs than the private sector [89]. Although there are many different proxies to measure organization size [90], following above studies [87,88,89], we adopt the total number of full-time employees to represent organization size and categorize organization property into four types: public institutions, state-owned companies, foreign companies, and private companies. And we incorporate these two variables as organization-level control variables in the model to eliminate their effects to the results. 

**Analytical approach.** Descriptive statistical analysis and correlation analysis were carried out with SPSS26.0 software (IBM, Armonk, NY, USA), and confirmatory factor analysis was performed with AMOS21.0 (IBM, Armonk, NY, USA). The subsequent direct effect test, mediating effect test, and moderating effect test were completed with HLM (Scientific Software International, Cambridge, MA, USA). Furthermore, the moderated mediation effect was tested using Mplus 7.0 (Muthen & Muthen, Los Angeles, CA, USA).

## 4. Results

### 4.1. Discriminate Validity

In this paper, the collected data at the individual level was subjected to confirmatory factor analysis (CFA) using AMOS21.0 (IBM, Armonk, NY, USA), and the model validity method was used to test the discriminate validity of the selected variables, as Table 3 shows.

The three-factor model fits very well with the data (RMSEA = 0.047, NNFI = 0.871, CFI = 0.933). The AIC (Akaike Information Criterion) is used to compare the baseline model (three-factor model) with the candidate model. The difference test between the measurement model and the candidate model shows that the three-factor model is significantly better than the alternative two-factor model and single-factor model. At the same time, by comparing the AIC values (the smaller the values are, the better the model), the three-factor model is also superior to the alternative model. Therefore, the above variables have good discriminant validity and are indeed four different constructs.

### 4.2. Common Method Biases Test

To avoid the possible impact of common method bias, this study still uses the Harman single factor method to test common method bias, through principal component analysis (political skill, subordinate relations, work-family conflict) using SPSS26.0 (IBM, Armonk, NY, USA). We found that the first factor explained 24.17% of the total variation, less than the standard of 40%, so we believe that the common method bias of the data is within the acceptable range [91].

### 4.3. Multicollinearity Test

We also centralized each variable and found that the tolerance of each variable is between 0.347 and 0.966, and the variance expansion factor VIF is between 1.035 and 2.878, far below the critical value of 10. Therefore, we believe that the model does not have a serious multicollinearity problem.

### 4.4. Correlation Analysis

Next, we performed some data-feature analysis on each variable: the mean (M), the standard deviation (SD), the correlation coefficient (r) of the main variables, and the reliability coefficient (α). The results of the analysis appear in Table 4. There is a significant correlation between the main variables of the model study. PS is positively correlated with SSGX (r = 0.377, *p* < 0.01) and negatively correlated with WFC (r = −0.367, *p* < 0.01); SSGX is negatively correlated with WFC (r = −0.324, *p* < 0.01). Therefore, it is assumed that H1, H2 and H3 can be initially verified, providing the necessary premise for testing the mediation effect of SSGX. In the end, the reliability analysis also showed that the Cronbach’s α coefficients of the four variables were all greater than 0.70 (the Cronbach’s α of FFPs is 0.957), consistent with the criteria that Fornell and Larcker (1981) propose, indicating that the variables have good internal consistency [92].

### 4.5. Hypotheses Testing

The independent variable of this paper is PS, and the dependent variable is WFC, from which the theoretical model is constructed. As Table 5 shows, we first put employee gender, employee age, employee education level, employee marriage status, whether an employee has children under 18 years old, and cooperation period between supervisor and subordinate in model M1 as control variables. 

Then, putting PS in model M2 and performing a regression test revealed that PS has a negative and significant impact on WFC (b = −0.118, *p* < 0.01), so Hypothesis 1 is supported. Next, model M7 uses SSGX as a dependent variable; putting PS into the model, the results show a positive and significant correlation between PS and SSGX (b = 0.420, *p* < 0.01), so Hypothesis 2 is also supported. In model M4 (b = −0.219, *p* < 0.01), the relationship between SSGX and WFC is significantly negatively correlated, so Hypothesis 3 is supported. In M5, putting FFPs and the control variables of organization size and organization property in level-2, we found that the coefficient of FFPs is significant (b = −0.360, *p* < 0.01), so we could do a further moderation test. In M6, the coefficient of interaction effect (SSGX *FFPs) is significant (b = 0.040, *p* < 0.01), which means the moderation effect of FFPs on the relationship between SSGX and WFC is supported (Hypothesis 5).

In order to test the mediation of SSGX, we adopted the Monte Carlo Method, and the result shows that the indirect value (a × b)’ 95% confidence interval (shown in Table 6) is [−0.113, −0.067], which does not include 0, so Hypothesis 4 is supported. Then, we used Mplus7.0 to test the moderated mediating effect of FFPs. Result showed that in the indirect effect test, the coefficient of difference between high and low FFPs is 0.037(*p* < 0.05), so Hypothesis 6 is supported.

**Figure 2:**
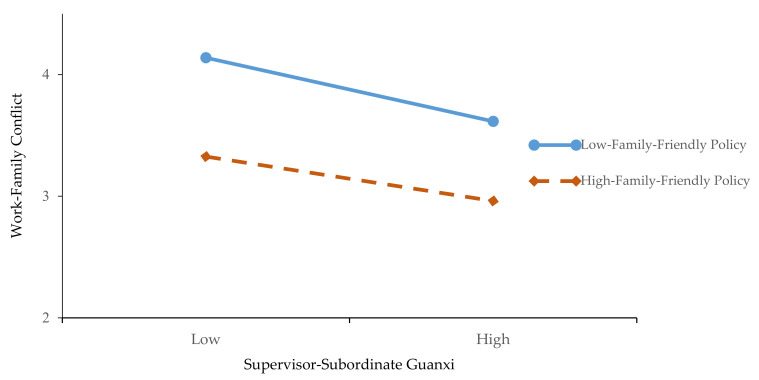
The Moderation Effect of Family-Friendly Policy on the Relationship Between Supervisor-Subordinate Guanxi and Work-Family Conflict.

**Figure 3:**
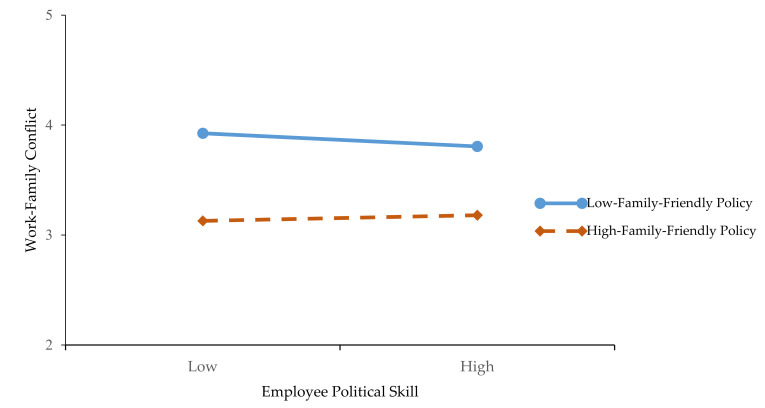
The Moderation Effect of Family-Friendly Policy on the Indirect Effect of Supervisor-Subordinate Guanxi.

Further, we use a simple slope method to draw the diagram of moderate effect. The low or high FFPs are obtained according to the average of variables to plus or minus one standard deviation. Figure 2 shows that the relationship between SSGX and WFC is stronger when FFPs are high than when they are low, and the results are consistent with Hypothesis 5. Figure 3 shows that the mediating effect of SSGX is stronger when the FFPs are high than when they are low, and the results are consistent with Hypothesis 6.

## 5. Discussion

### 5.1. Theoretical Implications

First, based on role theory, we found the negative effect of employee PS on WFC by obtaining work support and relieving role pressure. Antecedent variables affecting WFC mainly include working factors and individual factors [11]. Until now, few studies have focused on individual factors that can alleviate WFC. Although Jin et al. (2014) show in their research that individual coping skills, such as rational action and positive thinking, could alleviate individually perceived WFC [15], there is still a lack of follow-up research on the effect of individual skills on work-family balance. This research proved that employee PS can be a positive individual factor in alleviating WFC. This is not only another verification of role theory in the Chinese context but also an enrichment of the antecedent variables of WFC. Moreover, previous studies have examined the positive effect of employees’ PS on their promotion probability [21], career development [50] and performance rating [93]. This study extends the outcome of employee PS by proving the negative effect of PS on WFC.

Second, the paper verified the positive effect of employee PS on the establishment of SSGX. In previous studies, scholars tend to ignore the differences between the relationship in Eastern and Western contexts [66,77]. Although many scholars continue improving on the construction of Chinese SSGX [19,20], few empirical studies are based on the theoretical characteristics of SSGX, which are the key to explaining its influencing mechanism. Therefore, based on social exchange theory, the paper first analyzes the differences between SSGX and LMX, in terms of exchange scope, content, and status. SSGX forms mainly after work, and bases on emotional exchange. Because of the vertical status between the dyad, subordinate loyalty is the key to build and maintain close SSGX. We further discussed the positive role of PS in building SSGX in various dimensions according to the nature of SSGX. The research enriches the individual factor variables influencing the formation of Chinese Guanxi and reverified social exchange theory in the Chinese context.

Third, based on role theory and work-family spillover theory, as well as work/family border theory, the paper creatively explores the negative effect of SSGX on WFC and further tests the mediating effect of SSGX between employee PS and WFC. First, in close SSGX, supervisors would understand and accommodate their subordinates’ family life better and have a sense of “father-like” obligation to take care of their subordinates. The combination of role theory and “family-like” theory makes it easier for individuals to alternate their roles between work and family. These could all help employees reduce perceived WFC. Second, previous studies show that supervisor’s support could effectively alleviate employee perceived WFC [43]. In Chinese organizations dominated by differential patterns, supervisors would provide more support to “in-group” subordinates who are close to them [49], thus reducing the negative spillover of subordinates in the work [40,62], further alleviating their WFC. Finally, work/family border theory shows that the relationship between border-crossers (individuals who frequently move between work and family) and border-keepers (members who have particular influence on defining the border) could affect work-family balance [18,69]. However, empirical studies focusing on this topic are rare. From the perspective of SSGX, the study tests work/family border theory with empirical methods, verifying the negative effect of SSGX on WFC, and further testing the mediating effect of SSGX between employee PS and WFC. This paper explains the specific influencing mechanism of employees’ PS on their WFC, as well as verified work/family border theory in the Chinese context.

Fourth, the paper verifies the cross-level moderating effect of FFPs between SSGX and WFC, and the moderated mediating role of FFPs in the process of employee PS—> SSGX—> WFC. As organizational behavior theories do not show universality to a large extent, studies should be based on abundant situational information [94]. Work-family balance could benefit not only employees but also organizations [95], as the role of the organization is indispensable during the process of alleviating the employee’s WFC. From the perspective of substitution [83], the paper introduces FFPs, important context information from organization level, to build a more complete research model. When FFPs are high, employees could get family support both from supervisors and the organization’s official channels [80]. The substitute effect weakens the negative impact of SSGX on WFC, and vice versa. It indicates that the negative effect of SSGX on WFC has a boundary condition. Moreover, we discuss the moderated mediating role of FFPs—that is, that the mediation effect of SSGX between PS and WFC is weaker when organizations’ FFPs are high than when they are low. Briefly, the paper explores the specific influencing mechanism of PS on WFC at both individual and organizational levels, which makes the research results applicable to a larger extent.

### 5.2. Practical Implications

First, employees should pay attention to the significant role of PS in the workplace. Specifically, it is not only the key to career development [21] but also can help individuals reduce their perceived WFC, enabling employees to achieve success both in work and family. Different from political behaviors that may lead to negative results, PS is a kind of positive and effective social skill that could be cultivated in life practice. Ferris et al. (2005) proposed that employee PS levels could be effectively improved through systematic practical exercises, alternative learning, communication skills training, and drama methods [30]. Individuals should actively master these methods to improve their PS level. Also, from the organization’s perspective, those who are politically sensitive, proficient in interpersonal influence, and with extensive network resources tend to show lower levels of WFC, which is also beneficial to the development of the organization [23]. Liu et al. (2010) examined the learning effect of PS from the perspective of “teaching” and “learning”, pointing out that supervisors’ political teaching can help employees improve their PS [96]. Therefore, organizations should implement PS education programs to help employees improve PS and alleviate WFC.

Second, the influence of employee PS on WFC is realized by building good “Guanxi” with supervisors. Considering that SSGX plays an important role in Chinese organizations, individuals should accurately understand the connotation of SSGX and actively build good SSGX. Although previous studies show that family-supportive supervisor behavior could help employees reduce WFC [22], supervisors’ real primary goal is to efficiently complete team tasks [97]. Therefore, family-supportive behaviors of supervisors in organizations are still lacking [64]. Nevertheless, SSGX plays such a role in the Chinese context, and this characteristic Chinese-style relationship is formed outside of work and mainly built and maintained by employees’ active efforts, such as after-work visits, familiarization with leader’s family members, or spending time helping leaders deal with their personal chores [19,20]. Therefore, in a society oriented toward “Renqing” and “Guanxi”, employees must spend time and effort to actively use PS to build and maintain intimate SSGX, thus helping individuals reduce WFC.

Third, the influence intensity of SSGX on WFC is different amid high or low levels of organization FFPs. For instance, when FFPs provided by the organization are not enough, the negative effect of SSGX on employee perceived WFC becomes greater. Facing the positive role of SSGX and the family responsibilities that never disappear, employees will tend to invest more time and effort in building good relationships with their supervisors to reduce their WFC. Given the limited time and energy of employees, relationship-oriented behaviors eventually may harm the interests of the organization [98]. Therefore, although providing FFPs requires a certain cost [72], organizations should carefully weigh the input-output ratio of providing FFPs to ensure the maximum benefit. Undoubtedly, the organization’s policies and environments influence employee behaviors. Employees are individuals with subjective initiative [99]. They are not only recipients of organization policies; they also make corresponding judgments and adjust their behaviors according to different organizational environments [100]. When organizations provide higher FFPs, employees will save more time and energy for their job, which will ultimately achieve a win-win situation for both individuals and organizations.

### 5.3. Limitations and Future Research

Although empirical data has verified the theoretical hypothesis model and some innovative conclusions have been drawn, this study still has some limitations that suggest future research topics: 

First, although the common method deviation in this study is acceptable since the research data are time-lagged and multileveled [91]. However, common method deviation may exist in employees’ self-rating variables such as their PS, SSGX, and WFC. To respond to these, future research should broaden the channels and methods of data collection to avoid this problem to a greater extent. 

Second, although we used various methods to try to reach as many organizations as possible, due to the organization-employee matched design and the willingness of the employees, we only got 404 employee samples from 51 organizations, which is insufficient and may affect the accuracy of the judgment for all Chinese organizations [98]. Also, all the sample organizations have close ties to the authors. Therefore, the sample is not collected strictly according to the method of “random sample”. Expanding the sample size in future research is necessary, and conducting a “random sample” survey is advisable, if conditions permit.

Third, we controlled both individual and organizational variables that affect the research model, including employee’s education level, gender, age, marriage status, having children under 18, cooperation period between supervisor and subordinate, organization size and property. But unobservable characteristics, such as hidden motivations and preferences, may also cause endogeneity problem in this study [101,102]. From a design standpoint, our cross-sectional, nonexperimental design does not allow for definitive conclusions about causality, an issue to be tackled by including longitudinal data or using (quasi) experimental designs [103].

Fourth, “Guanxi” does play an important role in Chinese organizations. In addition to SSGX, individuals also have frequent and deep interactions with their team members in the workplace. According to Clark’s (2000) propositions, team members are domain members who also influence individuals’ WFC [18]. Therefore, future research could explore the role of team-member Guanxi in promoting work-family balance. Moreover, frequent supportive communication between employees and their family members about workplace activities could also alleviate the imbalance between work and family [69]. Hence, family relationship in the process of alleviating WFC is also an interesting topic worth further investigation.

Fifth, the study only tests the moderating role of organization FFPs in the mechanism of PS—>SSGX—>WFC. However, there may be other organizational- or individual-level variables, such as Guanxi human resource management practices and power distance, which may also function as boundary conditions. Future research can introduce more situational variables into this process to make research results more practical.

## 6. Conclusions

Many studies have examined the negative effects of work-family conflict on society, organizations, and individuals, yet, alleviating employee work-family conflict is worthy of more investigation. From personal skill and Guanxi perspectives, this paper finds that employee political skill could significantly lower perceived WFC, and SSGX plays the mediating role in this process. In addition, organization FFPs negatively moderate the relationship between SSGX and work-family conflict and moderate the indirect effect of employees’ political skill on their work-family conflict.

## Figures and Tables

**Table 1:** Differences between LMX and SSGX: Based on social exchange theory.

Type	Exchange Scope	Exchange Content	Status of Supervisor and Subordinate
LMX	In work	Instrumental exchange	Equal status
SSGX	Out of work	Emotional exchange	Emotional exchange

Note: refers to Law et al. (2000) [19], Chen et al. (2009) [20], and Sparrowe and Liden’s (1997) [44] studies combined with the authors’ understanding.

**Table 2:** Characteristics of the valid employee sample (*N* = 404).

Item	Category	Number	Percentage	Item	Category	Number	Percentage
Gender	Male	215	53.2	Work years in the organization	1 to 5	244	60.4
	Female	189	46.8		5 to 10	77	19.1
Age	Under 25	22	5.4		Above 10	83	20.5
	25 to 35	299	74.0	Cooperation periods between supervisor and subordinate	1 to 5	283	70.0
	35 to 45	62	15.3		5 to 10	92	22.8
	Above 45	21	5.2		Above 10	29	7.2
Education	Master’s or above	166	41.1	Marriage status	Single	133	32.9
	Bachelor’s	189	46.8		Married	271	67.1
	Junior college	46	11.4	Having kids under 18 years old or not	Yes	169	41.8
	High school or below	3	0.7		No	235	58.2

**Table 3:** Confirmatory factor analysis results of concept discrimination validity.

MODEL	*x* ^2^	*df*	*∆x* ^2^	AIC	NNFI	CFI	RMSEA
Three-factor model PS, WFC, SSGX	192.462	101	—	262.462	0.871	0.933	0.047
Alternative Two-factor model A PS + WFC, SSGX	367.756	103	175.294 **	433.756	0.753	0.807	0.080
Alternative Two-factor model B PS, SSGX + WFC	391.128	103	198.666 **	457.128	0.738	0.790	0.083
Alternative Two-factor model C PS + SSGX, WFC	429.152	103	236.69 **	495.152	0.712	0.762	0.089
Single-factor model PS + SSGX + WFC	579.773	104	387.311 **	643.773	0.611	0.653	0.107

Note: PS indicates political skill; SSGX indicates supervisor-subordinate Guanxi; WFC indicates work-family conflict; + represents two factors synthesized to one; ** *p* < 0.01.

**Table 4:** Mean, standard deviation and correlation coefficient of variables in level-1 ^a, b.^

Variables	M	SD	1	2	3	4	5	6	7	8
1 PS	3.613	0.461	(0.880)							
2 SSGX	3.799	0.475	0.377 **	(0.754)						
3 WFC	3.488	0.334	−0.367 **	−0.324 **	(0.705)					
4 GEN	0.468	0.500	−0.107 *	-0.023	0.053					
5 AGE	2.203	0.613	−0.053	0.010	0.016	0.046				
6 EDU	1.718	0.690	−0.090	0.044	−0.058	0.046	0.259 **			
7 MAR	0.671	0.471	−0.011	−0.043	0.022	0.002	0.370 **	0.042		
8 KID	0.582	0.494	−0.030	−0.033	0.017	−0.019	0.412 **	0.221 **	0.762 **	
9 LCO	2.371	0.615	−0.018	0.011	0.022	0.120*	0.419 **	0.230 **	0.261 **	0.407 **

Notes: ^a^ N = 404, * *p* < 0.05, ** *p* < 0.01; Political Skill (PS), Supervisor-Subordinate Guanxi (SSGX), Work-family Conflict (WFC); GEN means employee gender, males = 0, females = 1; AGE indicates employee age, below 25 = 1, between 25–35 = 2, between 35–45 = 3, above 45 = 4; EDU indicates employee educational level, master’s or above = 1, bachelor’s = 2, junior college = 3, high school or below = 4; MAR means marriage status, single = 0, marriage = 1; KID means having children under 18 years old or not, no = 0, yes = 1; LCO means cooperation period between supervisor and subordinate, less than 1 year = 1(removed from the final sample to represent stable SSGX), between 1–5 years = 2, between 5–10 years = 3, more than 10 years = 4; ^b^ The correlation coefficient is in the lower triangle of the matrix; the last column with () is the internal consistency coefficient α.

**Table 5:** Hypotheses 1, 2, 3 and 5 Tests.

Table. *Cont.*	WFC						SSGX
	M1	M2	M3	M4	M5	M6	M7
^a^ **Level-1**							
GEN	0.022	0.004	0.006	0.002	0.003	0.004	−0.037
AGE	−0.023	−0.025	−0.026	−0.024	−0.022	−0.022	−0.007
EDU	−0.005	−0.018	−0.001	−0.001	−0.003	−0.003	0.059
MAR	0.057 **	0.053 **	0.028 *	0.031 *	0.028	0.026	−0.125 *
KID	−0.002	0.002	0.006	−0.001	−0.003	0.003	0.074
LCO	−0.003	−0.002	0.011	0.008	0.009	0.009	−0.018
PS		−0.118 **	−0.019				0.420 **
SSGX			−0.211 **	−0.219 **	−0.215 **	−0.222 **	
^b^ **Level-2**						-	
SIZE				0.169 **	0.023	0.020	
PROP				0.116 **	−0.036	−0.036	
FFPs					−0.360 **	−0.367 **	
SSGX×FFPs						0.040 *	
σ^2^	0.022	0.017	0.009	0.010	0.009	0.010	0.159
τ_00_	0.089	0.090	0.090	0.054	0.013	0.013	−0.007
Pseudo-R^2^		0.036	0.108	0.423	0.802	0.793	

Notes: ^a^ Level-1 (N = 404); * means *p* < 0.05, ** means *p* < 0.01; Political Skill (PS), Supervisor-Subordinate Guanxi (SSGX), Work-Family Conflict (WFC); Control variables are employee gender (GEN), employee age (AGE), employee educational level (EDU), marriage status (MAR), having children under 18 years old or not (KID) and cooperation period between supervisor and subordinate (LCO); ^b^ Level-2 (*N* = 51); Family-friendly practices (FFPs); Control variables are organization size (SIZE), total number of full-time employees more than 1000 = 1, between 300 to 1000 = 2, less than 300 = 3; and organization property (PROP), public institutions = 1, state-owned companies = 2, foreign companies = 3, private companies = 4.

**Table 6:** Indirect effect of Supervisor-subordinate Guanxi.

Effect	Estimate	S.E.	Est./S.E.	p	95% CI
PS—>(a)SSGX	0.420	0.047	9.005	0.000	//
PS—>(b)SSGX—>WFC	−0.211	0.015	−13.969	0.000	//
Indirect effect	//	//	//	//	[−0.113, −0.067]

Note: All coefficients are normalized coefficients; *N* = 404; Political Skill (PS), Supervisor-Subordinate Guanxi (SSGX), Work-Family Conflict (WFC).
